# Gestational Diabetes Prevalence Estimates from Three Data Sources, 2018

**DOI:** 10.1007/s10995-024-03935-1

**Published:** 2024-05-29

**Authors:** Michele L.F. Bolduc, Carla I. Mercado, Yan Zhang, Elizabeth A. Lundeen, Nicole D. Ford, Kai McKeever Bullard, Denise C. Carty

**Affiliations:** 1 Office of Health Equity, Centers for Disease Control and Prevention (CDC), 2877 Brandywine Road, Atlanta, GA 30341 USA; 2 Division of Diabetes Translation, National Center for Chronic Disease Prevention and Health Promotion, CDC, Atlanta, USA; 3 Division of Reproductive Health, National Center for Chronic Disease Prevention and Health Promotion, CDC, Atlanta, USA; 4 Office of Women’s Health, CDC, Atlanta, USA

**Keywords:** Diabetes, Gestational, Pregnancy, Maternal health, Epidemiology, Birth certificates, Hospital records, Health surveys

## Abstract

**Introduction:**

We investigated 2018 gestational diabetes mellitus (GDM) prevalence estimates in three surveillance systems (National Vital Statistics System, State Inpatient Database, and Pregnancy Risk Assessment Monitoring Survey).

**Methods:**

We calculated GDM prevalence for jurisdictions represented in each system; a subset of data was analyzed for people 18–39 years old in 22 jurisdictions present in all three systems to observe dataset-specific demographics and GDM prevalence using comparable categories.

**Results:**

GDM prevalence estimates varied widely by data system and within the data subset despite comparable demographics.

**Discussion:**

Understanding the differences between GDM surveillance data systems can help researchers better identify people and places at higher risk of GDM.

## Significance

**What is Already Known on this Subject?:**

Gestational diabetes mellitus (GDM) prevalence varies by data system and population. Estimates of GDM prevalence are essential to inform prevention, identification, and management programs.

**What this Report Adds?:**

GDM prevalence estimates varied widely by data system (NVSS, SID, PRAMS) and participant demographics varied only slightly when a subset of comparable data were evaluated using jurisdictions available in all three systems (21 states and the District of Columbia). Understanding the differences between surveillance data systems can help researchers better identify people and places at higher risk of GDM.

## Objective

Characterized as an elevation of blood glucose concentrations first developed during pregnancy, gestational diabetes mellitus (GDM) is associated with pregnancy complications and adverse outcomes (Yang et al., 2006). People with GDM and their children are at higher risk of type 2 diabetes later in life (Bellamy et al., 2009; Dabelea et al., 2008). Some groups (e.g., older people, Asian people) are at higher risk of GDM and its complications (Li et al., 2020; Shah et al., 2021). Prevalence estimates for GDM can inform prevention, identification, and management programs; however, estimates may vary by data source. This paper presents GDM prevalence estimates from three data systems using (a) all available data in each system; and (b) data from a subset of people 18–39 years in 22 jurisdictions for which data were available in all three data systems. We additionally examined demographic characteristics of the analytic subset. An understanding of the differences between GDM surveillance data systems, including their strengths and limitations, can help researchers better identify people and places at higher risk of GDM.

## Methods

GDM prevalence was estimated using 2018 data from three surveillance systems: Centers for Disease Control and Prevention’s (CDC) National Center for Health Statistics’ National Vital Statistics System (NVSS) birth certificate data; Agency for Healthcare Research and Quality’s Healthcare Cost and Utilization Project State Inpatient Databases (SID) hospital discharge data; and CDC’s Pregnancy Risk Assessment Monitoring System (PRAMS) survey data.

Birth certificates are a complete enumeration of US births and are compiled in the NVSS.^1^ Birth certificates indicate the presence of GDM based on medical records. We queried the NVSS using CDC Wonder (https://wonder.cdc.gov/) for US states and the District of Columbia (DC). GDM prevalence was calculated as GDM-associated births divided by total live births, using only singletons or the first birth for multiple gestations to avoid overcounting people with multiple births.

The SID is an unweighted census of more than 95% of hospital discharge records at the state-level.^2^ Live births with administrative claims code(s) for GDM were identified using diagnosis-related group (DRG) and International Classification of Diseases, Tenth Revision, Clinical Modification (ICD-10-CM) codes.^3^ GDM prevalence was calculated for the available 27 states and DC, using total live hospital births with GDM divided by total live hospital births.

PRAMS is population-based survey of a sample of people with live births.^4^ Participants are sampled from birth certificates, with each participating jurisdiction sampling between 1,000 and 3,000 people with a recent live birth per year. PRAMS respondents self-report GDM during their most recent pregnancy. Weighted GDM prevalence and 95% confidence intervals (CI) for DC and 40 states with response rates > 50% in 2018 were calculated as births with GDM divided by total births among PRAMS respondents.

We also analyzed a subset of people for which data were available in all three data systems (limited to 22 jurisdictions and to ages 18–39 years due to small sample sizes of younger and older age groups) to examine demographic characteristics. All analyses were performed using SAS (version 9.4; SAS Institute), accounting for complex sampling and weighting in PRAMS.

## Results

**Fig. 1:**
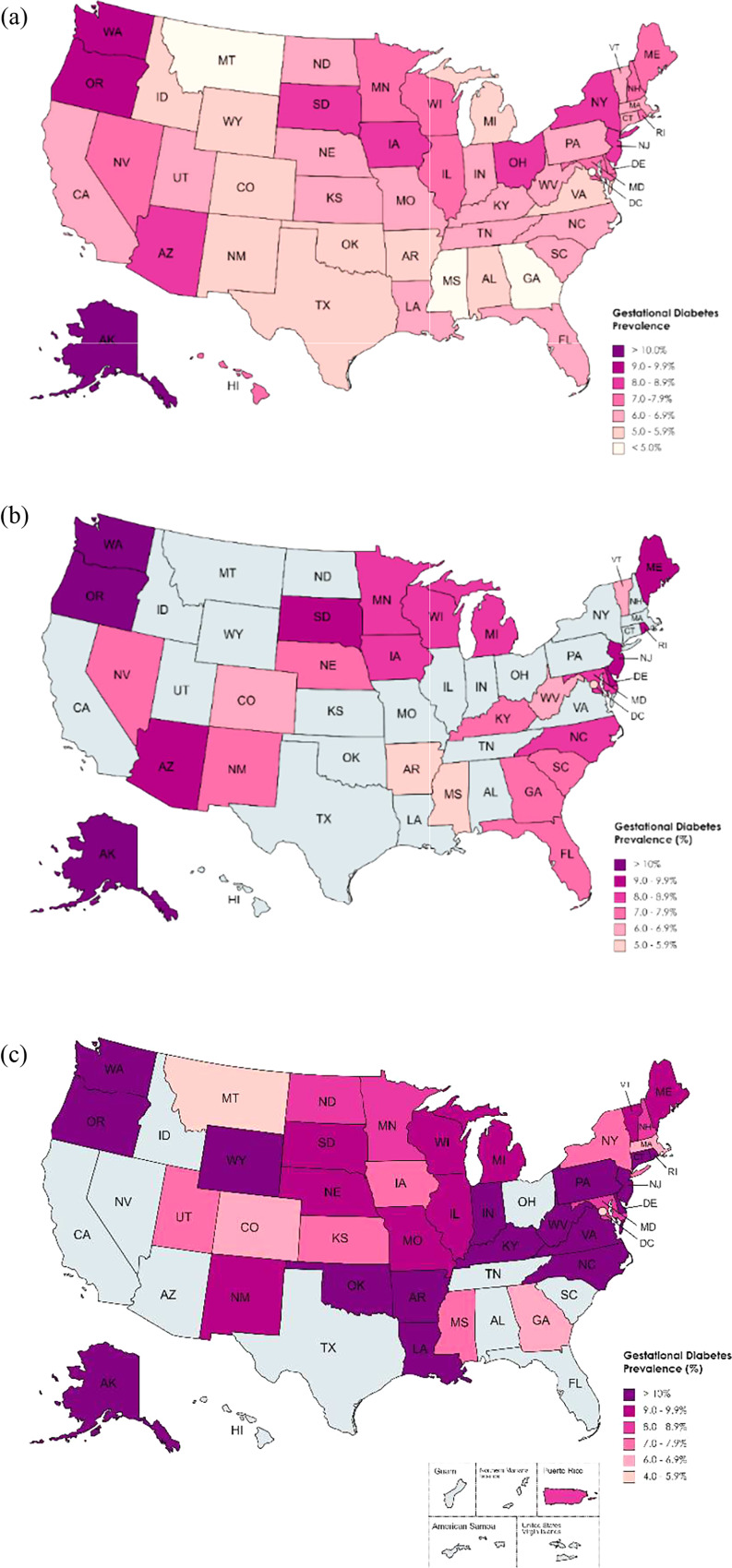
Gestational diabetes mellitus (GDM) prevalence estimates for 2018 as indicated by National Vital Statistics System (NVSS) (**a**), State Inpatient Database (SID) (**b**), and Pregnancy Risk Assessment Monitoring System (PRAMS) data (**c**). Greyed-out jurisdictions do not have data available for analysis. Maps created at mapchart.net

Using NVSS data for all states and DC, GDM prevalence ranged from 3.8% (Mississippi) to 11.0% (Alaska) [Fig. 1a]. Using the jurisdictions available in the SID, GDM prevalence ranged from 5.4% (Mississippi) to 13.2% (Alaska) [Fig. 1b]. In jurisdictions with data in PRAMS, prevalence ranged from 4.5% in DC (95% CI: 2.5–6.4%) to 13.8% in Alaska (95% CI: 11.4–16.2%) [Fig. 1c].

**Fig. 2:**
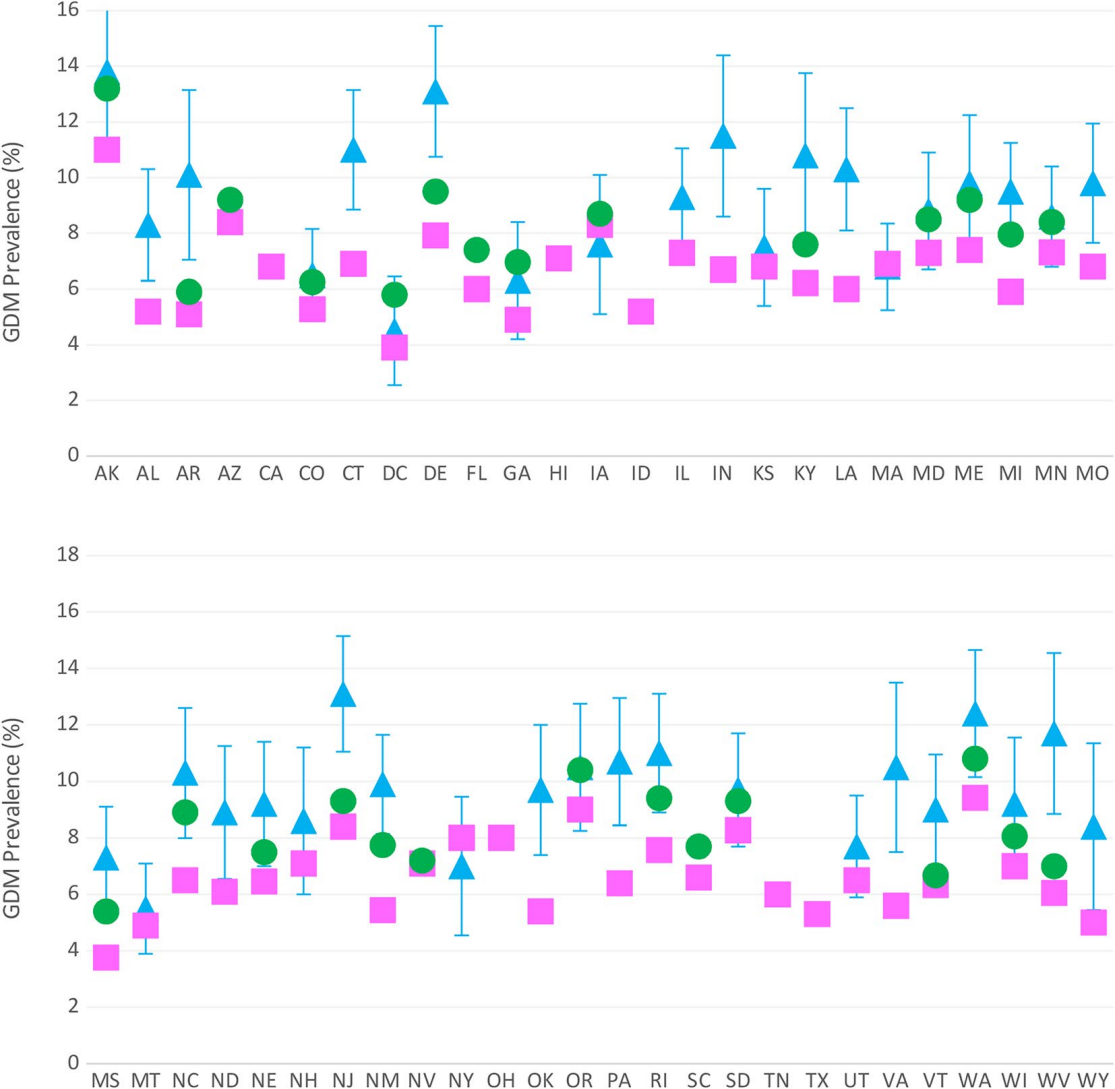
GDM prevalence estimates for 2018 for jurisdictions represented in National Vital Statistics System (pink square), State Inpatient Database (green circle), and Pregnancy Risk Assessment Monitoring System (PRAMS) (blue triangle). Blue error bars indicate 95% CIs for PRAMS estimates

GDM prevalence estimates varied widely by data system [Fig. 2]. Among 45 jurisdictions with data in more than one data system, discrepancies ranged from 0.1% (Massachusetts and Nevada) to 5.6% (West Virginia). Among 24 jurisdictions with data in all three systems, West Virginia had the largest range in prevalence (6.1–11.7%), while Colorado (5.3–6.4%) and Iowa (7.6–8.7%) had the smallest ranges across the three systems.

When analyzing subsets of people aged 18–39 years in the 22 jurisdictions for which data were available in all three systems, dataset-wide GDM still varied across the three data systems: 6.6% (NVSS), 8.0% (SID), and 9.0% (PRAMS; [95% CI: 8.5–9.6%]. Apart from race and Hispanic ethnicity, participant demographics were similar across data systems [Table 1]. A lower proportion of births in the NVSS were to non-Hispanic (NH) people of another race or more than one race compared with SID and PRAMS. A lower proportion of births in the SID were to Hispanic and NH Asian/Pacific Islander people compared to the other data systems. A higher proportion of births in PRAMS were to NH Black, NH White, and NH people of another race or more than one race. a higher proportion of births were missing race and ethnicity data in the SID (3.0%) and PRAMS (2.0%) compared to NVSS (0.7%).

**Table 1:** Demographic characteristics for people aged 18–39 years old who had a live birth in 22 Jurisdictions^a^ Represented in National Vital Statistics System, State Inpatient Database, and Pregnancy Risk Assessment Monitoring System, 2018

Characteristic^b^	National Vital Statistics System	State Inpatient Database	Pregnancy Risk Assessment Monitoring System
	*N* (%)	*N* (%)^b^	Weighted *N* (%)^c^ [95% CI]
Total Live Births	1,049,098	990,115	1,008,398
*Age, years*			
18–24	249,765 (23.8%)	235,252 (23.8%)	234,529 (23.3%) [22.4–24.2%]
25–29	323,385 (30.8%)	304,924 (30.8%)	308,994 (30.6%) [29.7–31.6%]
30–34	317,465 (30.3%)	300,173 (30.3%)	310,396 (30.8%) [29.8–31.7%]
35–39	158,483 (15.1%)	149,766 (15.1%)	154,480 (15.3%) [14.6–16.1%]
*Race and Hispanic Ethnicity* ^d^			
Hispanic	157,586 (15.0%)	123,568 (12.5%)	142,471 (14.4%) [13.8–15.0%]
Non-Hispanic	884,007 (84.3%)	836,509 (84.5%)	845,486 (85.6%) [85.0–86.2%]
American Indian, Alaska Native, Native American	11,741 (1.1%)	12,151 (1.2%)	10,954 (1.3%) [1.1–1.5%]
Asian or Pacific Islander	59,384 (5.7%)	49,317 (5.0%)	49,543 (5.9%) [5.5–6.3%]
Black	177,144 (16.9%)	170,748 (17.2%)	160,670 (19.0%) [18.1–19.9%]
White	611,425 (58.3%)	573,091 (57.9%)	588,168 (69.6%) [68.6–70.5%]
Another race/Multiple races	24,313 (2.3%)	31,202 (3.2%)	36,151 (4.3%) [3.9–4.7%]
Missing race or ethnicity	7,505 (0.7%)	30,038 (3.0%)	20,441 (2.0%)
*Location Classification* ^*e*^			
Metropolitan	849,437 (81.0%)	801,881 (81.0%)	813,912 (80.7%) [80.0–81.5%]
Nonmetropolitan	199,661 (19.0%)	187,906 (19.0%)	194,240 (19.3%) [18.5–20.0%]
Missing	0 (0.0%)	328 (0.0%)	246 (0.0%)
*Gestational Diabetes (GDM)*			
GDM prevalence	68,962 (6.6%)	79,328 (8.0%)	89,995 (9.0%) [8.5–9.6%]
Missing data on GDM	1,149 (0.1%)	0 (0.0%)	11,182 (1.1%)

^a^The jurisdictions included are Alaska, Arkansas, Colorado, District of Columbia, Delaware, Georgia, Iowa, Kentucky, Maryland, Michigan, Minnesota, Mississippi, North Carolina, New Jersey, New Mexico, Oregon, Rhode Island, South Dakota, Vermont, Washington, Wisconsin, and West Virginia. Maine and Nebraska are also available in all three data systems for 2018; however, in SID, we were not able to identify 18-39-year-olds in Maine with age categories, and Nebraska is missing race data, so these two states were excluded from the table

^b^SID totals calculated as crude percentages using unweighted totals across the 22 jurisdictions

^c^PRAMS prevalence (95% CI) estimates account for complex sampling using weights and appropriate survey procedures

^d^SID is categorized by ethnicity, and non-Hispanic people were further categorized by single-race and more than one race. SID also combines Asian and Pacific Islander people in one group. PRAMS data for Other Non-White and Mixed Race were combined for the Another Race/Multiple Race category

^e^Metropolitan/nonmetropolitan classification was determined for all datasets using the 2013 NCHS Urban-Rural Classification Scheme for Counties

## Discussion

We document variation in GDM prevalence estimates in three data systems, and these variations exist even when comparing subsets of participants with similar demographic characteristics. GDM prevalence estimates appear to be influenced by the strengths and limitations of each data system, including representativeness of the data, varying demographic and geographic coverage, recall bias for surveys, and completeness of documentation in administrative data.

NVSS data provide a complete enumeration of live births in US states and territories and contain detailed race and ethnicity information. However, NVSS may underreport GDM; studies have documented a low sensitivity relative to medical records (46–75.7%) (Gregory et al., 2019; Dietz et al., 2015; Devlin et al., 2009). Data quality also varies across hospitals (Gregory et al., 2019). Further studies might explore how to improve documentation of GDM on birth certificates.

SID data have several strengths, including that they encompass more than 95% of US community hospital discharges. However, jurisdiction participation changes over time.^5^ In addition, SID is derived from claims data, which are subject to coding errors (e.g., missing and inaccurate data). Further, unlike NVSS and PRAMS, the SID only includes hospital births. While NVSS indicates that < 2% of births nationwide occurred outside of a hospital in 2018, some juridictions have higher rates of non-hospital births, such as Alaska (7%). Pregnancies affected by GDM are at increased risk of adverse outcomes and may require hospital-based management during delivery, which may result in higher prevalence of GDM in the SID than might be seen in other birth settings. South Dakota, Rhode Island, West Virginia, and DC had more hospital deliveries identified in the SID than live births in birth certificate data—for unknown reasons. Finally, SID has fewer race and ethnicity groupings relative to the NVSS and PRAMS, limiting our results to Hispanic and non-Hispanic single race categories.

PRAMS offers detailed survey data on health before, during, and after a live birth, which are linked to demographic data in birth certificates. However, jurisdiction participation in the survey varies year to year.^6^ In addition, survey response rates vary by jurisdiction and have been decreasing over time; seven states participating in 2018 did not meet the 50% response rate criteria. PRAMS may also be subject to biases associated with self-reported data (DeSisto et al., 2014; Dietz et al., 2014). Nevertheless, a 2014 validation study of PRAMS data found moderate sensitivity and excellent specificity, but poor positive predictive values for self-reported GDM, compared to medical records, indicating that self-report of GDM may indeed be a valid information source (Dietz et al., 2014).

This study has two primary limitations. First, it carries over the limitations of the original data systems. Second, we caution against comparing GDM prevalence estimates across the data systems, because each uses different methodologies and includes different populations.

Nevertheless, this study provides a description of GDM prevalence data from three data systems and considers their strengths and limitations as data sources for GDM surveillance.

GDM screening combined with high-quality surveillance data can inform public health efforts to prevent, identify, and manage this potentially serious pregnancy complication. Improved surveillance, such as through hospital-based quality improvement initiatives, is needed to increase the accuracy of the data and to better identify the disproportionate burden of GDM across geographic and demographic factors. However, all three data systems can provide a useful source of information for prevention and management of GDM. Each could be used by public health practitioners to increase availability of prevention programs to groups disproportionately impacted by GDM, such as the National Diabetes Prevention Program for which people with a history of GDM are eligible.

## Data Availability

NVSS data used for this project are publicly available via CDC Wonder (https://wonder.cdc.gov/natality.html). Access to HCUP and PRAMS datasets must be requested by researchers.

## Footnotes

1 https://wonder.cdc.gov/natality.html.

2 https://hcup-us.ahrq.gov/db/state/siddbdocumentation.jsp.

3 If Diagnosis-Related Group (DRG) version is 35, then DRG codes 767–768 or 774–775 for vaginal delivery and 765–766 for C-section were used; if DRG version is 36, then DRG codes 768 or 796–798 or 805–807 for vaginal delivery and DRG codes 783–788 for C-section were used. Live births were identified using International Classification of Diseases, Tenth Revision, Clinical Modification (ICD-10-CM) codes delivery with at least one live birth (Z37.0, Z37.2, Z37.3, Z37.5, Z37.6). Births with documented GDM were identified by ICD-10 codes O24.4 or O99.81 listed anywhere on the discharge record. ICD-10 codes for pre-pregnancy diabetes were excluded (E08, E09, E10, E11, E13, O24.0, O24.1, O24.3, O24.8, or O24.9).

4 https://www.cdc.gov/prams.

5 https://hcup-us.ahrq.gov/db/availability_public.jsp.

6 https://www.cdc.gov/prams/prams-data/researchers.htm#response.
